# Association between hypomineralization of deciduous and molar incisor hypomineralization and dental caries

**DOI:** 10.1590/0103-6440202204807

**Published:** 2022-08-26

**Authors:** Yasmy Quintero, Manuel Restrepo, Diego Fernando Rojas-Gualdrón, Aline Leite de Farias, Lourdes Santos-Pinto

**Affiliations:** 1 Department of Morphology, Genetics, Orthodontics and Pediatric Dentistry, São Paulo State University (Unesp), Araraquara School of Dentistry, Araraquara, São Paulo, Brazil.; 2 Basic and Clinical Research Group in Dentistry, School of Dentistry, CES University, Medellín, Colombia.; 3 School of Medicine, CES University, Medellín, Colombia.

**Keywords:** Dental care, dental caries, dental enamel, molar incisor hypomineralization, primary teeth

## Abstract

This study aimed to evaluate the association between the severity of hypomineralized second primary molars (HSPM), molar-incisor hypomineralization (MIH) and dental caries in children. 450 children between the ages of 6 and 7 years were included in this cross-sectional study. A calibrated examiner classified the enamel hypomineralizations and dental caries lesions using the MIH and HSPM and the Nyvad criteria, respectively. The primary outcome was the severity of MIH according to the severity of HSPM. Statistical analysis was performed using the generalized linear model and ordinal logistic regression. The prevalence of concomitant MIH and HSPM was 26% sex and age adjusted. Mild enamel defects were more frequent than severe enamel defects. An association was found between the severity of MIH and HSPM, both for mild defects (OR=87.54; 95%CI: 55.87, 137.17) and severe defects (OR=82.15; 95%CI: 45.72, 147.61). The severity of hypomineralization in permanent molars was associated with the activity of dental caries lesions (OR=29.85; 95%CI: 12.95, 68.83). To conclude, there is a strong association between the severity of HSPM and MIH, which is more significant in the presence of active dental caries lesions.

## Resumo

O objetivo desse estudo foi avaliar a associação entre a severidade da Hipomineralização de Segundos Molares decíduos (HSMD), da Hipomineralização de Molares e Incisivos (HMI) e cárie dentária em crianças. Neste estudo transversal foram incluídas 450 crianças entre 6 e 7 anos de idade. Um examinador calibrado classificou as hipomineralizações e lesões de cárie dentária utilizando o índice da HMI/HMD e o critério Nyvad, respectivamente. O desfecho primário foi a severidade da HMI de acordo com a severidade da HSMD. As análises estatísticas foram realizas usando o modelo linear generalizado e regressão logística ordinal. A prevalencia concomitante da HMI e HMSD foi de 26 % ajustada por sexo e idade. Defeitos leves foram mais frequentes que os defeitos severos. Foi encontrada a associação entre a severidade da HMI e da HSMD para defeitos leves (OR=87.54; IC95%: 55.87, 137.17) e severos (OR=82.15; IC95%: 45.72, 147.61). A severidade da hipomineralização em molares permamentes foi associada a atividade da lesão de cárie dentária (OR=29.85; IC95%: 12.95, 68.83). Conclui-se que existe uma forte associação entre a severidade da HSMD e da HMI, a qual foi mais significante na presença lesões ativas de cárie.

## Introduction

Demarcated enamel hypomineralizations can occur in primary and permanent teeth. In 2001, Weerheijm, Jälevik, and Alaluusua proposed the term “molar-incisor hypomineralization (MIH)” to describe a hypomineralization of systemic origin that affects one to four first permanent molars and that is frequently associated with permanent incisors ^1^. Later, in 2008, Elfrink et al proposed the term “hypomineralized second primary molars (HSPM)” ^2^. Clinically, MIH and HSPM share the same clinical features, such as demarcated opacities ranging from creamy-white to yellow-brown in color, posteruptive enamel breakdown, and atypical dental caries lesions/restorations. Also, it is not unusual to find other primary or permanent teeth with similar defects ^3^. Currently, the etiology of MIH and HSPM is considered multifactorial, with the possible influence of local, systemic, genetic, and environmental factors ^4^ ^,^ ^5^.

In a recent systematic review and meta-analysis, Garot et al compared the prevalence of MIH in patients with HSPM against healthy individuals ^6^. The authors showed that HSPM is associated with a higher probability of presenting MIH (OR= 4.66; 95% CI: 2.11, 10.26) and concluded, based solely on the presence/absence of the defects, that HSPM is a predictive sign of MIH ^6^. However, the association between HSPM and MIH in terms of severity has not been investigated.

The association between dental caries and enamel hypomineralization has also been demonstrated ^7^ ^,^ ^8^. A systematic review showed that the probability of finding a child with MIH and dental caries is 2.1-4.6 times higher than finding a child with MIH without dental caries.^7^ Alterations in the structure and composition of hypomineralized teeth, such as increased porosity, higher carbon and carbonate content, and the presence of posteruptive enamel breakdown, have been proposed to explain this association ^9^. However, the relationship between hypomineralization and the activity of the dental caries lesion has been poorly investigated.

In terms of diagnosis, prognosis, treatment plan, monitoring, and communication with those responsible for the patient, it is pertinent to establish the severity of the hypomineralizations of the first permanent molars in the presence of HSPM and its connection with dental caries activity. Thus, this study aimed to evaluate the association, at tooth level, between the severity of MIH and HSPM. A secondary objective was to explore the potential moderator effect of the dental caries activity on this association.

## Material and methods

This study is reported following the STROBE (Strengthening the Reporting of Observational Studies in Epidemiology) statement.

### Study design

This cross-sectional study was approved by the Human Ethics Committee of CES University (Act #127, 2018) and followed the principles of the Declaration of Helsinki. Furthermore, the patients provided assent to participate in the study, and their parents signed the informed consent.

### Setting

This study was conducted in six randomly selected schools in Medellin (Colombia), according to the information provided by the local Education Secretariat. The data were collected between June 2018 and November 2019.

### Participants

Four hundred and fifty children between 6 and 7 years of age, born and residing in Medellin, with at least two second primary molars and two first permanent molars, were included. Patient selection was made through convenience sampling (invitation to three public schools and three private schools in Medellin). Children with syndromes or medical conditions related to developmental enamel defects (DED) were excluded.

### Variables

For each tooth, the severity of MHI was the dependent variable, and the severity of HSPM was the independent variable. Dental caries activity was considered a potential effect variable. Age and sex were considered confounding variables.

### Data sources and measurements

The clinical examination was conducted in a school environment, following the recommendations by the World Health Organization for conducting epidemiological studies regarding the organization of personnel, instruments and supplies, infection control, examination area and position, and lighting ^10^.

The demarcated enamel hypomineralizations in primary and permanent teeth were evaluated using the MIH and HSPM index, proposed and validated by Ghanim et al ^11^ ^,^ ^12^. This index considers the eruption stage, the clinical presentation of the defect, and the extension of the MIH and HSPM. It also allows differentiating these hypomineralizations from other developmental defect of enamel (DDE), such as diffuse opacities, hypoplasias, and amelogenesis imperfecta ^11^. The severity of the MIH and HSPM was classified as mild when the tooth presented color change only and as severe when it presented posteruptive enamel breakdown, atypical dental caries lesion/restoration, or had been extracted ^13^.

The dental caries lesions were evaluated according to the Nyvad criteria, ^14^ which are a validated visual-tactile criterion used to evaluate the activity and severity of dental caries lesions ^14^ ^,^ ^15^ ^,^ ^16^. The Nyvad criteria include sound surfaces, surfaces with active or inactive non-cavitated, microcavitated or cavitated lesions, and filled surfaces ^14^.

### Bias

The demarcated enamel hypomineralizations were evaluated following the recommendations by Elfrink et al ^17^ and the MIH and HSPM index training manual ^13^. The dental caries lesions were evaluated following the recommendations by Nyvad and Baelum ^16^.

The clinical examination was performed by a single experienced examiner, who was previously calibrated for the use of the MIH and HSPM index and the Nyvad criteria. A reference examiner calibrated the examiner for the diagnosis and classification of DDE and dental caries lesions. For this purpose, a set consisting of 60 photographs, including different degrees of severity of MIH and HSPM and dental caries lesions, was used. The examiner evaluated the photographs twice with an interval of one week between evaluations. The results were compared with the reference examiner, obtaining an intra- and inter-examiner reliability through the Kappa coefficient. The intra- and inter-examiner Kappa coefficients for the MIH and HSPM were 0.75 and 0.70, respectively. The intra- and inter-examiner Kappa coefficients for the dental caries lesions were 0.90 and 0.88, respectively.

### Study size

The sample size was estimated to test the null hypothesis, i.e., the equality of MIH odds ratio according to the presence or absence of HSPM (Ho: OR = 1). Assuming an MIH prevalence of 5% in the presence of HSPM, an HSPM prevalence ≥ 14%, and a strength of association (OR) ≥ 2.7, a sample of at least 450 participants was needed to obtain a ≥ 80% power with a 5% precision. The expected value for the OR was taken as a reference because it was higher than the lower limit of the confidence interval (CI) of the association reported by da Silva-Figueiredo et al. (OR= 6.31; 95% CI: 2.6, 15.1) ^18^.

### Statistical methods

The children’s distribution by age and sex was estimated using frequencies and percentages. At the patient level, the prevalence of MIH, HSPM, and dental caries was estimated as the percentage of children with at least one affected tooth among the total number of children included. At the tooth level, primary and permanent teeth distribution was estimated using frequencies and percentages. The tooth-level prevalence of MIH, HSPM, and dental caries was estimated as the percentage of teeth with at least one affected surface among the total number of teeth. Additionally, the distribution of the severity of MIH and HSPM was presented.

The association between the severity of MIH and HSPM was analyzed at tooth level for the pairs of primary and permanent teeth by quadrants. For this, the ordinal logistic regression model was utilized, which allows analyzing associations with ordinal dependent variables, such as the severity of MIH. The strength of association adjusted for age and sex was estimated using OR and a 95% confidence interval and *P*-value. Cluster-robust variance estimation was used to determine autocorrelation between primary and permanent teeth pairs within patients. Finally, to explore the potential moderating effect of the presence and activity of dental caries on the association between the severity of MIH and HSPM, a subgroup analysis was performed by including the variable ‘dental caries’ in interaction with the independent variable ‘HSPM’. Estimates of the strength of association between MIH and HSPM in healthy teeth and teeth with active and inactive dental caries were presented. The model adjustment was analyzed with the link test; goodness of fit was assessed with the Akaike Information Criteria (AIC); the McFadden pseudo-R^2^ is also presented.

The analyses were conducted using Stata (College Station, TX, USA), version 16. The significance level was set at *P* < 0.05.

## Results

### Participants

Of 591 eligible children, 141 were excluded, which resulted in a sample of 450 children. Of these, 63.55% were 7 years old, and 50.22% were male. In total, 3,399 teeth were evaluated, of which 1,784 (52.5%) were primary and 1,616 (47.5%) were permanent.

### Descriptive data

The prevalence of HSPM at the patient level was 23.78% (95% CI: 19.73, 27.82), and at the tooth level was 4.04% (95% CI: 3.59, 4.54). The prevalence of dental caries was 65.11 % (95% CI: 60.59, 69.62). In the primary dentition, the prevalence of dental caries at the patient level was 62.89% (95% CI: 58.31, 67.46), and at the tooth level was 19.32% (95% CI: 19.39, 20.22). In the permanent dentition, the prevalence of dental caries at the patient level was 12.89% (95% CI: 9.68, 16.09), and at the tooth level was 2.41% (95% CI: 1.96, 2.96).

Table 1 shows the severity of hypomineralizations by tooth type. One hundred and two children (22.7%) presented MIH and HSPM, 11 (2.4%) MIH, 4 (0.9%) HSPM, and 333 (74.4%) did not present MIH or HSPM.

### Outcome data

The prevalence of MIH at the patient level was 25.11% (95% CI: 20.99, 29.22), and at the tooth level was 10.92% (95% CI: 9.99, 11.97). The permanent tooth most frequently affected by MIH was the mandibular right first molar (2.7%), and the primary tooth most frequently affected by HSPM was the mandibular right second molar (5.1%).

**Table 1:** Severity of molar-incisor hypomineraliation in permanent (MIH) and primary (HSPM) teeth by quadrants.

Quadrant	Tooth	MIH severity			Tooth	HSPM severity		
		Healthy	Mild	Severe		Healthy	Mild	Severe
Upper right	16	346 (86.3)	47 (11.7)	8 (2.0)	55	396 (89)	37 (8.3)	12 (2.7)
Upper left	26	330 (85.1)	49 (12.6)	9 (2.3)	65	397 (89.2)	33 (7.4)	15 (3.4)
Lower left	36	349 (84.3)	55 (13.3)	10 (2.4)	75	380 (85.2)	45 (10.1)	21 (4.7)
Lower right	46	333 (80.8)	68 (16.5)	11 (2.7)	85	386 (86.2)	39 (8.7)	23 (5.1)

### Main results

Table 2 shows the association of the severity of HSPM with the severity of MIH adjusted by age and sex. We observed a strong association in the severity of MIH according to the severity of HSPM, both for mild defects (OR= 87.54; 95% CI: 55.87, 137.17) and severe defects (OR= 82.15; 95% CI: 45.72, 147.61). This association was not influenced by age or gender. The prevalence of concomitant MIH and HSPM was 26% (95% CI: 21.83, 30.16).

**Table 2:** Sex-and-age adjusted association of the severity between HSPM and MIH.

	OR	***P*-value**	95% CI
Severity of HSPM			
Healthy*	1.0		
Mild	91.7	<0.001	58.1, 144.6
Severe	86.0	<0.001	47.6, 155.4
Sex			
Female*	1.0		
Male	0.8	0.214	(0.6, 1.1)
Age			
6 years*	1.0		
7 years	1.3	0.233	(0.9, 1.9)

* Reference category

### Other analyzes

Table 3 shows the association of the severity of HSPM with the severity of MIH adjusted by age and sex, according to the activity of dental caries lesions in the second primary molars. This strength of association was considerably higher when the second primary molars had an active dental caries lesion (mild defects, OR= 379.79; 95% CI: 62.87, 2292.87. Severe defects, OR= 370.37; 95% CI: 73.04, 1878.10). The interaction term between HSPM and the activity of dental caries lesions in the second primary molars was statistically significant (p-value = 0.0133)

Regarding the association between the activity of dental caries lesions and MIH and HSPM, an association was observed between active lesions and hypomineralization, being more significant in permanent teeth (OR= 29.85; 95% CI: 12.95-68.83) than in primary teeth (OR= 1.79; 95% CI: 1.26, 2.56). No significant association was found between inactive lesions and MIH (OR= 1.81; 95% CI: 0.88, 3.71) or HSPM (OR= 0.71; 95% CI: 0.45, 1.13).

This final model including HSPM, activity of the dental caries, age and sex had no specification error (link test, p-value = 0.707) and explained 41.4% of the variability in MIH severity according to McFadden pseudo-R^2^, with an AIC = 979.7 (compared to an AIC = 1634.8 for the null model).

**Table 3:** Sex-and-age adjusted associations of the severity between HSPM and MIH, according to the activity of dental caries lesions in the second primary molars.

	OR	***P-*value**	95% CI
Severity of the HSPM			
	SPM with no dental caries lesion		
Healthy *	1	-	-
Mild defect	88.0	<0.001	52.2, 148.5
Severe defect	35.9	<0.001	15.1, 85.1
	SPM with active dental caries lesion		
Healthy *	1	-	-
Mild defect	379.7	<0.001	62.9, 2292.8
Severe defect	370.4	<0.001	73.0, 1878.1
	SPM with inactive dental caries lesion		
Healthy *	1	-	-
Mild defect	74.33	<0.001	7.91, 698.12
Severe defect	Not estimable		

Abbreviations: SPM, second primary molar

* Reference category

## Discussion

This study shows that there is a strong association between the severity of MIH and HSPM. Also, the activity of the dental caries lesion plays a crucial role since it influences the strength of this association and the severity of the defects.

In recent years, an increase in the prevalence of DDE has been reported in both primary and permanent teeth. Our findings regarding the prevalence of concomitant HSPM and MIH (26%) resemble the average reported in the systematic review and meta-analysis by Garot et al (27.60%) ^6^. However, it differs from the individual average of HSPM and MIH by 12.61% and 10.2%, respectively ^6^. A discrepancy in the prevalence of these types of DDE among different populations can be expected due to geographic differences and exposure to distinct environmental factors. In addition, health-related factors, genetics, and lifestyle can affect the amelogenesis and the clinical presentation of HSPM and MIH ^4^ ^,^ ^19^.

Regarding severity, mild defects were the most common in both primary and permanent teeth, which supports the results of other studies ^2^ ^,^ ^20^. However, severe defects were observed in higher proportions in the second primary molars than the first permanent molars. This finding can be explained by the age of the patients (6-7 years) included in this study, whose primary molars have been present for a longer period than the permanent molars. Also, longitudinal studies have shown that demarcated enamel hypomineralization tend to be more severe as age increases, as they relate to posteruptive enamel breakdowns, dental caries lesions, and restorations ^21^ ^,^ ^22^. Thus, it is crucial that the diagnoses of HSPM and MIH are made at 3-5 years ^17^ ^,^ ^23^ and 8 years ^(^ ^3^ of age, respectively.

HSPM is a predictive sign of MIH. Children with HSPM are up to 4.66 more likely to present MIH than children without HSPM ^6^. Our findings, in addition to sustaining the association between HSPM and MIH, show, for the first time, that there is a strong association in terms of severity. That is, the greater the severity of HSPM, the greater the severity of MIH. Thus, for the prediction of MIH, it is essential to consider not only the presence/absence of HSPM ^6^, but also the severity of the defect in the second primary molar. The crown formation of the second primary molar and the first permanent molar concur from birth to approximately the tenth month of life. Hence, the presence of a risk factor in this period could affect both types of teeth. The possible influence of prenatal, perinatal, and postnatal factors on HSPM and MIH has been discussed in the literature.^5^ However, a causal factor has not been established, and therefore, they are considered DDE of multifactorial, polygenic origin, and to be influenced by the environment ^4^ ^,^ ^5^.

According to the results of this study, the activity of dental caries lesions in the second primary molar significantly impacts the strength of association and severity of enamel hypomineralizations. This means a greater probability of finding active lesions than inactive caries lesions in patients with severe defects. Alterations in the structure and composition of hypomineralized teeth, such as increased porosity, higher carbon and carbonate content, and the presence of posteruptive enamel breakdown, have been proposed to explain the association between dental caries and enamel hypomineralization ^9^.

Our results are relevant for the clinician because they reinforce the importance of early diagnosis of DDE in the primary dentition. These findings also help establish a prognosis and plan preventive strategies and monitoring to avoid complex and costly restorative procedures for the patient and those responsible. Besides, they support the results of other studies that have demonstrated the association between MIH and DDE in other permanent teeth ^24^ ^,^ ^25^. In terms of severity, de Farias et al reported that mild hypomineralizations in the second permanent molar were more frequent when the first permanent molar presented severe MIH (OR= 4.01; 95% CI: 2.50-7.77) ^24^.

Among the strengths of this study, we highlight the use of validated criteria for HSPM and MIH and dental caries, examiner calibration, and the inclusion of a representative sample size, which favors internal and external validity. Likewise, the design and reporting followed the recommendations by Elfrink et al for the preparation of standardized studies on this subject, which allows future comparisons ^17^. One of the limitations of this study is that it is impossible to determine the causes of the association between the severity of HSPM and MIH due to its cross-sectional design. Furthermore, the age of the patients could have influenced the severity of the defects. Lastly, the estimated associations between the severity of HSPM and MIH according to the activity of dental caries lesions produced wide, unprecise confidence intervals for the OR because of the small subgroup sample size. However, the statistical test for the interaction term confirmed the heterogeneity of the association depending on the activity of dental caries lesions. A prospective design, i.e., a cohort study, could tackle these limitations; however, it should be considered that this type of study requires more time and a larger number of participants due to losses to follow-up and subgroup analyses ^6^.

In conclusion, there is an association between the severity of HSPM and MIH, which is considerably stronger in the presence of active dental caries lesions.
